# School Refusal Behavior in Japan: The Impact of COVID-19 on Children

**DOI:** 10.3390/children12091105

**Published:** 2025-08-22

**Authors:** Daisuke Matsubara, Kazuhiko Kotani, Hitoshi Osaka

**Affiliations:** 1 Division of Community and Family Medicine, Jichi Medical University, Tochigi 329-0498, Japan; 99081dm@jichi.ac.jp; 2 Department of Pediatrics, Jichi Medical University, Tochigi 329-0498, Japan; hosaka@jichi.ac.jp

**Keywords:** childhood, COVID-19, disparity, mental health, wellbeing

## Abstract

School refusal behavior, defined as a child’s prolonged voluntary absence from school for reasons unrelated to illness and/or economic hardship, is a growing concern in Japan. The COVID-19 pandemic has worsened this issue by disrupting children’s lives. This review summarizes the prevalence, contributing factors, and health implications of school refusal, particularly in the context of COVID-19. A literature review of government reports and PubMed-indexed studies indicates that school refusal in Japan has been rising for eleven years, reaching a record 340,000 cases in 2023. Middle school students (6.7%) were the most affected, followed by elementary school students (2.1%). The pandemic intensified school-related, family-related, and child-related risk factors. School closures disrupted routines, reduced peer interactions, and increased social isolation, contributing to higher rates of anxiety and depression. Reports of suicides and mental health disorders among children have also surged. Family stressors, including economic hardship and parental mental health struggles, further exacerbate school refusal. Additionally, remote learning has widened socioeconomic disparities in access to education, leaving vulnerable children at greater risk. Addressing school refusal requires a multifaceted approach involving schools, families, healthcare providers, and policymakers. School-based interventions, mental health approach, and flexible educational programs would be essential. The Japanese government’s “COCOLO Plan” represents progress toward a more inclusive education system, and a comprehensive, interdisciplinary strategy is needed. Ensuring all children receive the necessary support to reengage with education is critical to overcoming the long-term challenges posed by school refusal.

## 1. Introduction

Japan is facing a unique challenge of a super-aging society with a low birth rate, possibly reflecting future trends in many other countries [1]. The annual number of births in Japan has been declining for nine consecutive years, reaching its lowest level in 2023 with total fertility rate of 1.20 [1]. Interestingly, in contrast to the declining number of children, school refusal behavior is a growing issue in Japan and worldwide [2,3]. Defined as a child’s prolonged voluntary absence from school for reasons unrelated to physical illness and/or economic reasons, it has had social and psychological implications [3]. While various factors contribute to school refusal behavior, the COVID-19 pandemic has amplified this by affecting children’s health, social structures, and educational experiences [2]. The impact of COVID-19 on this status indicates the points necessary for the future plan, which should be reviewed in the period when we live with COVID-19. In spite of the significance of school refusal, this topic has been rarely reviewed in Japan. Thus, this review summarizes the important points such as the prevalence, contributing factors, and health implications of school refusal in Japan, particularly in relation to COVID-19.

We conducted a literature review using available government reports and peer-reviewed studies that were searched via PubMed until 31st December 2024. The focus to select literature was on school refusal behavior and its relationship with the COVID-19 pandemic. While this manuscript aims to summary the situation in Japan, many key sources were published in Japanese. For English-language studies, we thoroughly selected those that were thematically relevant to the focus of this manuscript. In addition, several Japanese studies provided references to international trend, which helped to contextualize Japan’s situation and could aid to establish a deeper understand of this essential topic in other countries. In this way, this narrative review was made. A summary of references we selected is provided in Table 1.

## 2. The Current Status of School Refusal Behavior in Japan and Worldwide

The Ministry of Education, Culture, Sports, Science and Technology in Japan has reported an increasing number of school refusal cases in middle and elementary school for consecutive eleven years since 2013 [2]. In Japan, school refusal is defined as students who are voluntarily absent from school for over 30 days for reasons unrelated to physical illness and/or economic reasons. The number showed a gradual increase until 2020, but in 2021, during the COVID-19 pandemic, the number showed a sharp increase, reaching over 240,000 cases (2.6%). Its impact remains, reaching over 340,000 cases (3.7%) in 2023, which is the highest recorded number to date. Among them, absent school days of 30–49, 50–89 and over 90 days are 22.4%, 22.7% and 55.0%, respectively. In addition, 4.2% of students experiencing school refusal have not been properly consulted or managed. School refusal is prevalent among middle school students (5.0% in 2021 to 6.7% to 2023), followed by elementary school students (1.3% in 2021 to 2.1% in 2023). The reasons for school refusal include “unmotivated toward school” (32.2%), followed by “anxious or depression mood (23.1%)” and “disturbed daily life (23.0%)” in both elementary and middle schools [2]. These findings could highlight the profound impact of the pandemic on school children.

As there is no clear consensus on the duration of absence that constitutes school refusal, it can be challenging to estimate the actual number of children affected by school-related issues. Similar trends have been observed globally. In the United States, chronic absenteeism nearly doubled during the pandemic, with rates reaching 28.3% among K-12 students [4]. In the United Kingdom, emotion-based school avoidance has risen, now affecting over 22% of students, approximately double the pre-pandemic rate [5]. In 71 lower-middle and high-income nations, the overall population-weighted prevalence of chronic school absenteeism was 11.4% [6]. Globally, the pandemic can influence children’s behavior toward school. Economic hardships and limited access to technology created further barriers to education, often preventing children from returning to school after the pandemic.

## 3. Factors Contributing to School Refusal

School refusal arises from the complex interplay of multiple factors [3,5]. Several methods have been proposed to categorize these factors. For instance, Lester and Michelson suggested three primary categories: school-related, family-related, and child-related factors [5]—which we adopt in this manuscript.

School-related factors include bullying, peer problems, academic difficulties, academic pressures, and poor home-school relationships. Family-related factors encompass parents’ physical and mental health problems, divorce or separation, family conflict, and the presence of young carers. Child-related factors include physical and mental health issues, special educational needs, neurodevelopmental disorders, behaviorally inhibited temperament, and low self-esteem [5].

In Japan, family-related problems, such as parent–child relationship, are a major concern among elementary school students, whereas school-related issues, such as poor academic performance or peer problems, predominate among secondary school students [2]. Notably, child-oriented problems such as lethargy or anxiety were significant concerns for both groups, affecting approximately 30% of students. All or some of these factors may have been affected or exacerbated by the negative impact of the COVID-19 pandemic on children across biological, psychological, and social dimensions [5,7].

## 4. The Impact of COVID-19 on Children

The COVID-19 pandemic has profoundly affected children’s lives, with impacts on physical health, mental wellbeing, family dynamics, and societal structures [4,5,6,7]. These changes are thought to be associated with school refusal by intensifying the existing challenges and creating new barriers.

### 4.1. Physical and Mental Health

COVID-19 has altered children’s physical and psychological wellbeing [4,5,6,7,8]. Prolonged school closures and lockdowns have disrupted daily routines, leading to increased screen time and reduced physical activity. Physical fitness scores among Japanese children have declined, with many Japanese children experiencing sleep disturbances and weight gain due to sedentary lifestyles [8]. A study from Japan found that school closures resulted in elementary and junior high school students spending more time with their families and sleeping, but their sleep rhythms, eating habits, and physical activities were disrupted [9]. Concurrently, mental health disorders, such as anxiety and depression, have surged. A survey revealed that among Japanese middle and high school students, the number of those who show a depressive mode increased from 6.4% in 2020 to 13.3% in 2023, exacerbated by isolation, loss of extracurricular activities, and uncertainty about the future [10]. Japanese children’s suicides, including those in elementary, middle, and high schools, had remained stable at approximately 300–350 cases per year since 2011. But in 2020, during the COVID-19 pandemic, the number increased sharply, exceeding 500 cases annually in 2022 and 2023 [11].

Globally, the prevalence of mental health disorders in children and adolescents has also increased during the COVID-19 pandemic. A meta-analysis revealed that 1 in 4 youth (age of <18 years) experience clinically elevated depression symptoms, while 1 in 5 youth experience clinically elevated anxiety symptoms, which are double the pre-pandemic estimates [12]. Another cross-sectional study of Canadian youth and young adults (age of 6–20 years) found an increase in anxiety with incidence rate ratio (IRR) of 1.11, personality disorders with IRR of 1.21, and suicidality with IRR of 1.10 in females and an increase in eating disorders in both sexes (IRR of 1.66 in females, IRR of 1.47 in males) in the COVID-19-prevalent period [13]. This trend also applied to suicide in children. A systematic review on emergency department visits for all indications among children and adolescents (aged < 19 years) across 18 countries revealed good evidence of an increase for attempted suicide during the pandemic (rate ratio 1.22, 90% Confidence interval (CI) 1.08–1.37), modest evidence of an increase for suicidal ideation (rate ratio 1.08, 90% CI 0.93–1.25), and good evidence of only a slight change in self-harm (rate ratio 0.96, 90% CI 0.89–1.04) [14].

Social isolation during the pandemic further deepened feelings of loneliness and anxiety, affecting children’s ability to form and maintain friendships. A study in Japan suggested that COVID-19-related sleep disorders affected feelings of school refusal through the exacerbation of loneliness directly or indirectly [15]. Although the long-term outcome of mental health disorders in children is unclear, the prolonged stress of the pandemic may have contributed to neuroinflammatory changes, influencing the hypothalamic–pituitary–adrenal axis [7].

### 4.2. Family Dynamics

The pandemic’s economic and emotional toll has strained family relationships, creating challenges in providing a supportive environment for children. Parents, particularly mothers, reported heightened stress levels during school closures in Japan [16]. Another study in Japan showed that approximately 40% of parents experienced moderate depression symptoms during the COVID-19 pandemic [10], while this trend was reported similarly in other countries as well [12,17]. An international meta-analysis found that clinically significant depression symptoms and anxiety symptoms during the COVID-19 pandemic were found in 26.9% and 41.9% of mothers of children aged 0–5 years [12]. Clinically elevated depression and anxiety symptoms were more prevalent in older mothers, and clinically elevated anxiety was more common in highly educated mothers [12]. Notably, pre-pandemic vulnerabilities are at higher risk of negative outcomes than those without. A cross-sectional online survey in the United Kingdom suggested that parents with children who requires special needs are at higher risk of loneliness, parent burnout and decreased perception of social support [18]. In addition, a longitudinal study in the Netherlands found that parents with higher levels of negative emotions prior to lockdown were more likely to perceive COVID-19-related stress [19].

Although considerable numbers of parents experienced elevated levels of depression and/or anxiety initially at the pandemic, symptoms often decreased over time. For instance, a two-wave longitudinal study on Norwegian parents found that depression meeting clinical criteria decreased from 23% in the first wave to 16.8% in the second wave, similarly anxiety meeting clinical criteria decreased from 23.3% to 13.8% [20]. While many parents show resilience as a common outcome due to COVID-19 pandemic, family dynamics should be considered in the context of children’s health and should be a target of future interventions [21].

Economic instability disproportionately affects low-income families, limiting their ability to access resources necessary for remote learning. The reduced availability of social services and school-based support systems has further compounded the difficulties faced by vulnerable families. Increased domestic tensions during lockdowns have led to an increase in Japanese reports of domestic violence (190,030 cases in 2020, increasing approximately 1.6 times higher than in 2019) and child abuse (225,509 cases in 2023, a 5.0% increase compared to 2022), further destabilizing children’s home environments [22,23]. These familial factors could profoundly affect the child–parent relationship, which in turn may positively or negatively affect children’s mental wellbeing states [24].

### 4.3. Societal Impacts

On a broader scale, the COVID-19 pandemic has exposed and exacerbated societal inequalities, particularly in education. Prolonged school closures and lockdowns due to the COVID-19 pandemic may have contributed to these educational disparities [25]. A paper from a World Bank report by Patrinos described the educational impact of the pandemic on school children [26]. The duration of school closures in Japan was over 10 weeks, varying across countries from 0 weeks (Sweden) to over 90 weeks (India). The report by Patrinos estimated that for every week schools were closed, learning levels declined by almost one percent of a standard deviation—meaning that a 20-week closure could reduce leaning outcomes by 0.20 standard deviation, equivalent to nearly one year of schooling [26].

Socioeconomic status also contributed educational gaps during the pandemic, particularly in lower-income countries [25]. Remote learning highlighted disparities in access to technology and digital infrastructure, among children from socioeconomically disadvantaged households [27]. These disparities widened the achievement gap and limited the educational opportunities of students [28]. Since the duration of lockdown and school closures was determined based on public health decisions, we should examine the link between these decisions and their spillover effects—not only on learning outcomes but also on broader human capital factors—which could influence children in various ways.

The impact of the pandemic extended to children’s social development, as restrictions on group activities and social gatherings reduced opportunities for meaningful interactions with peers. An oversea study investigating changes in developmental screening scores among American children of 0–5 years of age between the pre-pandemic period (1 March 2018, to 29 February 2020) and the intra-pandemic period (1 June 2020, to 30 May 2022) found significant declines in the communication (−0.029), problem-solving (−0.018), and personal-social (−0.016) domains [29].

### 4.4. Direct Impact on School Refusal

The COVID-19 pandemic has intensified the factors contributing to school refusal. For students already struggling with academic pressure, remote learning environments can lack necessary structure and support, increasing students’ feelings of frustration and disengagement. Fear of infection and concern about safety also discouraged many students from returning to in-person schooling, especially those with health vulnerabilities or at-risk family members [30].

The COVID-19 pandemic has had a particularly severe impact on students who are already vulnerable to difficulties in school attendance. These children, including those with pre-existing mental health conditions, neurodevelopmental disorders, or adverse family environments, face heightened challenges due to the disruptions caused by the pandemic [30,31]. A large-scale cross-sectional online survey conducted during the COVID-19 pandemic in Hong Kong, involving families with children aged 2–12 years, found that the risk of child psychosocial problems was higher in children with special educational needs, acute/chronic disease, mothers with mental illness, single-parent families, and low-income families [32]. Another questionnaire survey in Japan of caregivers of children with neurodevelopmental disorders showed that although no relationship was observed between difficulty in infection prevention measures and deterioration in their relationship with parents and friends during school closures, a positive association was found after school reopened [33].

The COVID-19 pandemic has revealed many aspects of social vulnerability, particularly among children in vulnerable situations, which had previously remained unrecognized. The lessons we have learned from this pandemic could serve to prepare for future pandemics and to create a more inclusive society.

## 5. Interventions, Support, and Future Directions

Addressing school refusal behavior and its associated challenges requires a multifaceted approach. School-based interventions such as counseling services, peer support programs, and teacher training in mental health awareness are essential for identifying and supporting at-risk students [2,5,27]. Schools should foster inclusive environments that accommodate diverse needs and ensure that all students feel valued and supported.

Family support plays a relevant role in addressing school refusal. Providing resources and education to parents can help mitigate family-related stressors. Parenting workshops and family therapy can address the underlying dynamics and strengthen relationships, fostering a supportive home environment for children [34].

At the societal level, government policies must prioritize mental health services and educational equity. Ensuring equal access to technologies and educational opportunities is vital for reducing disparities [35]. Telemedicine and digital tools, which have gained attention during the pandemic, offer valuable options for providing mental healthcare to children who are reluctant to attend in-person consultations [27].

In 2023, the ‘COCOLO Plan (Comfortable, Customized and Optimized Locations of learning)’ was implemented by the Japanese government as a comprehensive, multi-sector approach to address school refusal in Japan [35]. This plan set forth three clear goals: (1) securing diverse learning opportunities, (2) early detection and support through the “one device per student” initiative, and (3) visualizing school culture to make schools places where “everyone can learn with ease”.

The plan promotes support not only for children experiencing school refusal but also for their families by integrating several measures. These include promoting the establishment of in-school education support centers (such as special support rooms), assigning school counselors and school social workers, developing an environment in which students can easily consult with such specialist at any time via online services, supporting education through ICT or collaborating with Non-Profit Organizations and free schools.

It also emphasizes early detection of changes in health and feelings of students through individual devices and early-stage interventions by “team schools” to identify the support needs of students facing difficulties.

This plan is expected to expand various educational options for children experiencing school refusal. For instance, under certain conditions, students attending free schools or learning through ICT-based methods may have their attendance officially recognized. This could bring Japan more in line with international best practices to support diverse learning needs—for example, the U.S Department of Defense Education Activity, which officially recognizes homeschooling as a valid alternative to school attendance [36].

To address the complex and evolving issues of school refusal, future research should focus on several key areas—such as longitudinal studies to evaluate the long-term mental health, educational, and social outcomes of children and families; comparative international studies to examine how different policies/systems/cultures influence school refusal; and biopsychosocial approaches that integrate biological, psychological, and social factors affecting children.

## 6. Conclusions

The COVID-19 pandemic has impacted school refusal behavior in Japan and worldwide in various ways. Its effects have been pronounced with pre-existing vulnerabilities and those from socially disadvantaged backgrounds. Addressing this issue requires coordinated efforts to provide support through schools, families, and societal systems.

School refusal arises from the complex interplay of multiple contributors, often making it difficult for children to recognize underlying problems. While this can be a challenging experience, it also presents an opportunity for a deeper understanding and stronger relationships for both children and their families. Physicians, healthcare providers, and the government play a crucial role in supporting these children and their families in overcoming these challenges together, just like a team and community, so that they can move toward a future.

## Figures and Tables

**Table 1:** Summary of references selected in the review.

Authors (Ref. No.)	Year	Summary
Ministry of Health, Labor, and Welfare, Japan (Ref. [1])	2023	Japan’s official annual demographic statistics highlighting a record low total fertility rate of 1.20 in 2023, which showed the demographic backdrop against which school refusal is increasing.
Ministry of Education, Culture, Sports, Science, and Technology, Japan (Ref. [2])	2023	Japan’s official annual report on school absenteeism, including details on prevalence, reasons for absenteeism, and the sharp increase during COVID-19, with breakdowns by school level and absence duration.
Kearney CA (Ref. [3])	2008	A review article defining school absenteeism and refusal behaviors in youth, which discussed prevalence, etiological factors, and frameworks for intervention, forming the conceptual basis for the review.
Dee TS (Ref. [4])	2024	A report from the Proceedings of the National Academy of Sciences of the United States of America, describing chronic absenteeism trends in U.S. schools, showing a near doubling during the pandemic to 28.3%.
Lester KJ, Michelson D (Ref. [5])	2024	A review paper, exploring the phenomenon of emotionally based school avoidance in the post-COVID-19 era, which highlighted the ‘perfect storm’ of risk factors exacerbated by pandemic disruptions.
Rahman MA et al. (Ref. [6])	2023	A large multi-country study on chronic absenteeism in 71 nations, examining prevalence and associated factors.
de Figueiredo CS et al. (Ref. [7])	2021	A review paper exploring the multifactorial impact of COVID-19 on children’s and adolescents’ mental health, which integrated biological, environmental, and social influences relevant to school refusal.
Ministry of Education, Culture, Sports, Science, and Technology, Japan (Ref. [8])	2021	Japan’s official annual report tracking physical fitness trends among Japanese children, which noted declines during COVID-19 due to reduced physical activity and disrupted routines.
Saito M et al. (Ref. [9])	2022	An original article assessing mental health in Japanese schoolchildren during school closures, which found disrupted sleep, eating habits, and physical activity patterns.
National Center for Child Health and Development, Japan (Ref. [10])	2023	Japan’s national center report summarizing the overall health impact of COVID-19 on Japanese children, which included mental, physical, and lifestyle changes observed during the pandemic.
Ministry of Health, Labor and Welfare, Japan (Ref. [11])	2024	Japan’s official annual report on child suicide in Japan, which highlighted a sharp increase in suicides among children during the pandemic.
Racine N et al. (Ref. [12])	2021	A meta-analysis of global studies on depression and anxiety in children and adolescents during COVID-19, which found doubled prevalence compared to pre-pandemic estimates.
Roumeliotis N et al. (Ref. [13])	2024	A Canadian population-based study reporting increased youth hospitalizations for mental health issues during the pandemic, which highlighted conditions such as anxiety, personality disorders, and suicidality.
Madigan S et al. (Ref. [14])	2023	A systematic review and meta-analysis of pediatric ED visits for suicide attempts, self-harm, and ideation, which found significant increases during the pandemic.
Okajima, I et al. (Ref. [15])	2022	An original article from Japan linking COVID-19-related sleep problems and anxiety to loneliness, which identified an indirect link to school refusal feelings among adolescents.
Hiraoka D, Tomoda A (Ref. [16])	2020	An original article from Japan on parenting stress during school closures, which found heightened stress linked to disrupted routines and caregiving demands.
Bourion-Bédès S et al. (Ref. [17])	2023	An original article from France examining stress levels in parents during the first lockdown, which identified socioeconomic and family structure factors as risk modifiers.
El-Osta A et al. (Ref. [18])	2021	A UK cross-sectional survey on the mental health of parents during lockdown, which reported elevated depression and anxiety symptoms linked to childcare and homeschooling burdens.
Achterberg M et al. (Ref. [19])	2021	A Dutch longitudinal study on the mediation effect of perceived stress on parental and child wellbeing, which showed pre-existing negative emotions increased vulnerability to pandemic stress.
Johnson MS et al. (Ref. [20])	2021	A Norwegian longitudinal study following parents during and after the first lockdown, which found declines in clinically significant depression and anxiety over time, suggesting resilience in some families.
Whaley GL, Pfefferbaum B (Ref. [21])	2023	A review article summarizing parental challenges during the pandemic, which discussed risk and protective factors relevant to child outcomes.
Gender Equality Bureau Cabinet Office, Japan (Ref. [22])	2021	Japan’s official white paper on gender equality, including statistics showing a significant increase in domestic violence reports during COVID-19.
Children and Families Agency, Japan (Ref. [23])	2023	Japan’s official annual report on child abuse cases, noting a 5% increase in 2023 compared to 2022, with implications for child mental health.
Cohodes EM et al. (Ref. [24])	2021	An original article from the U.S. on parental buffering of stress during COVID-19, which found that supportive parenting can mitigate youth psychological symptoms despite pandemic stressors.
Agostinelli F et al. (Ref. [25])	2022	An economic analysis on the role of schools, peers, and parents in child development during closures, highlighting the unequal effects of shutdowns across families.
Patrinos HA (Ref. [26])	2023	A World Bank analysis quantifying learning loss per week of school closures, which estimated that long closures can result in learning deficits equivalent to nearly one school year.
Goudeau S et al. (Ref. [27])	2021	A review article on how COVID-19 lockdown and remote learning increased the social class achievement gap, which explained mechanisms by which inequalities in resources and support widened.
Nippon Foundation and Mitsubishi UFJ Research and Consulting, Japan (Ref. [28])	2021	A survey assessing the impact of COVID-19 on educational disparities in Japan, which found significant gaps in access to technology and learning resources.
Johnson SB et al. (Ref. [29])	2024	An original article from the U.S. showing developmental score declines in children aged 0–5 years during COVID-19, which affected communication, problem-solving, and personal-social skills.
McDonald B et al. (Ref. [30])	2023	A UK qualitative study on school attendance problems post-pandemic, which captured perspectives of parents and professionals on re-engagement challenges.
Dessain A et al. (Ref. [31])	2024	A systematic review of longitudinal cohort studies on children with ADHD during the pandemic, which found worsening mental health symptoms and functional difficulties.
Tso WWY et al. (Ref. [32])	2022	A Hong Kong survey of families during COVID-19, which identified greater psychosocial risk in children with special educational needs or from vulnerable families.
Yamamoto T et al. (Ref. [33])	2022	A Japanese caregiver survey on children with neurodevelopmental disorders during school closures, which found links between infection prevention difficulties and relationship changes post-reopening.
Matano M et al. (Ref. [34])	2022	A pilot study from Japan on internet-delivered parent–child interaction therapy for children with ASD, which suggested feasibility of remote intervention during pandemic restrictions.
Ministry of Education, Culture, Sports, Science, and Technology, Japan (Ref. [35])	2023	A Japanese government’s ‘COCOLO Plan’ addressing school refusal, which outlined goals for diverse learning opportunities, early detection, and inclusive school environments.
Department of Defense Education Activity (Ref. [36])	2025	Information from the U.S. DoDEA recognizing homeschooling as a valid alternative to formal school attendance, which provided an international comparison for Japan’s evolving policies.

## Data Availability

The authors declare that data supporting the findings of this study are available in the article.
